# Orientation Probability and Spatial Exogenous Cuing Improve Perceptual Precision and Response Speed by Different Mechanisms

**DOI:** 10.3389/fpsyg.2017.00183

**Published:** 2017-02-08

**Authors:** Syaheed B. Jabar, Britt Anderson

**Affiliations:** ^1^Department of Psychology, University of Waterloo, WaterlooON, Canada; ^2^Centre for Theoretical Neuroscience, University of Waterloo, WaterlooON, Canada

**Keywords:** attention, probability learning, visual perception, spatial cuing, orientation discrimination

## Abstract

We are faster and more accurate at detecting frequently occurring objects than infrequent ones, just as we are faster and more accurate at detecting objects that have been spatially cued. Does this behavioral similarity reflect similar processes? To evaluate this question we manipulated orientation probability and exogenous spatial cuing within a single perceptual estimation task. Both increased target probability and spatial cuing led to shorter response initiation times and more precise perceptual reports, but these effects were additive. Further, target probability changed the shape of the distribution of errors while spatial cuing did not. Different routes and independent mechanisms could lead to changes in behavioral measures that look similar to each other and to ‘attentional’ effects.

## Introduction

Probability effects are easy to induce. When some target stimuli occur more often than others, we react to them more quickly and report them more accurately. This probability benefit occurs in simple detection tasks (Laberge and Tweedy, 1964; Miller and Pachella, 1973; Hon et al., 2013), in visual-search tasks (Wolfe et al., 2007; Rich et al., 2008), and in perceptual estimation tasks (Anderson, 2014; Jabar and Anderson, 2015). These behavioral outcomes resemble the effects of typical ‘attentional’ exogenous spatial cuing (e.g., Posner and Cohen, 1984) where cued targets are also reacted to more quickly and reported more accurately. As such, probability and spatial cuing might be suspected to reflect a common ‘attentional’ locus (e.g., Hon and Tan, 2013).

While attentional cues and probability biases both result in response facilitation, there are differences. For example, Wyart et al. (2012) and Cheadle et al. (2015) showed that they could distinguish probability-cuing of target identity from trial-by-trial attentional cuing when using a reverse correlation procedure. One possibly important methodological detail is that in these studies the probability information was explicitly provided to participants, as opposed to our work where participants are naive to the manipulation and remain so throughout the experiment (as verified by post-experiment questionnaires; Anderson, 2014; Jabar and Anderson, 2015). It could be that on-line, implicit probability learning leads to faster and more precise estimates of the orientation of probable stimuli by the same mechanisms as spatial cuing (Anderson and Druker, 2013), but that additional and distinct mechanisms contribute to behavioral benefits when information is explicitly provided.

On the other hand, we have previously found that the effects of orientation and spatial probability are dissociable even when they were both implicitly driven (Jabar and Anderson, in press), supporting the idea that the similar performance benefits of probability and spatial cuing may only be superficial. Examining both effects within a single perceptual estimation task would provide us with information as to whether or not this is the case. If the two manipulations interact, as is the case with attentional and memory manipulations (Liu and Becker, 2013; Haskell and Anderson, 2016), they likely share the same mechanism. If they create additive, independent effects, that provides support for the idea that underlying mechanisms of orientation probability and spatial exogenous cuing are separable.

The current study combines the designs of Anderson and Druker (2013) and Anderson (2014). While those experiments tested orientation probability and exogenous spatial cuing separately, here we conjointly manipulated both in an effort to demonstrate if there are distinct underlying mechanisms.

## Materials and Methods

### Participants

Twenty participants (*median* age = 19 years) were recruited from the University of Waterloo (16 females, 4 males), in exchange for course credits. Nineteen reported themselves right-handed. All participants had normal or corrected-to-normal vision. This study was approved by the University’s Office of Research Ethics.

### Stimuli

**FIGURE 1:**
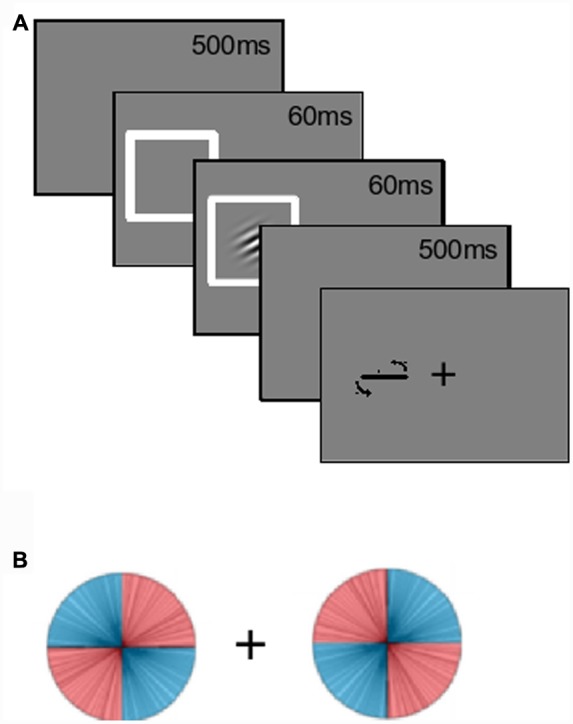
**Experiment paradigm. (A)** Participants fixated onscreen for 500 ms. The left or right location was spatially cued for 60 ms, then a Gabor appeared in one of the two locations. The (uninformative) cue and Gabor offset following an additional 60 ms. After a delay of 500 ms, a response line appeared for participants to make their estimations. **(B)** Gabor orientations were manipulated based on location. For example, when the Gabor appeared on the left, it was more likely (80%) to be left-tilting. This was reversed on the right. The location-orientation conjunction was counterbalanced across participants.

Gabors were used as the target stimuli. The Gabors were oriented grayscale sine-wave gratings with a circular Gaussian mask (**Figure 1A**), shown at 50% maximum contrast with an average measured luminance of 39 cd/mm^2^. The Gabors had a spatial frequency of four cycles per degree of visual angle, and were presented on a gray background with a similar luminance of 40 cd/mm^2^. When viewed from a distance of 60 cm, the Gabors subtended approximately 4° of visual angle both vertically and horizontally. Gabors appeared either left or right of the black fixation cross, with a distance of 4° from the center of the cross to the center of the Gabor. Lines, used as feedback and for participants to rotate to report their estimations, had a length of four visual degrees and always occurred in the same location as the Gabor for that trial. Response lines always started off horizontal.

Gabors were equally likely to appear at each location. Only one Gabor was presented on each trial. *Collapsed across these two locations*, all orientations were equally likely. The critical manipulation was the occurrence-rate of the *probability-location conjunctions*. Half the participants saw the conjunction depicted in **Figure 1B**: When a Gabor appeared on their left, its orientation was more likely to be left-tilting, and this was reversed on the right. Tilts were uniformly distributed across each quadrant. High-probability orientations accounted for 80% of the trials. The lines in **Figure 1B** depict the distribution observed by the first participant. The location-orientation conjunctions were counterbalanced across participants.

Probability distributions were maintained throughout the experiment. For every set of 20 trials, there were eight left-tilting Gabors on the left, eight right-tilting Gabor on the right, etc. Participants were *not* informed about these probability distributions. Practice trials had uniformly distributed orientations. Spatial exogenous cuing was done by having a white (72 cd/mm^2^) box surround the Gabor location. These cues were uninformative (50% congruent with Gabor location, 50% incongruent), and there was always one cue on each trial. A gamma-corrected CRT monitor that refreshed at 89 Hz was used, and stimulus timings were programmed as numbers of frames (*mean* refresh = 11.27 ms, *SD* = 0.07 ms).

Auditory feedback was given after each response to maintain motivation. A high pitched sound indicated an error of less than 12°. A lower pitch ^1^ indicated an error greater than 12°. Participants were not informed of the exact error threshold.

### Procedure

Prior to the task, participants were instructed to make their estimations of the Gabor orientations as accurately as they could. They were *not* told that they needed to be fast. Responses were made with a QWERTY keyboard using their dominant hand.

Participants were also instructed to fixate on the central fixation symbol at the start of each trial. This fixation phase lasted 500 ms. A spatial cue then appeared at either the left or right location for approximately 60 ms (five screen refreshes), after which a Gabor appeared. The cue and the Gabor disappeared together approximately 60 ms later (again five frames). After a delay of 500 ms a response line appeared. Participants made their orientation estimations by rotating this line counter-clockwise or clockwise by pressing “Z” or “C” on the keyboard. This rotation was at a maximum of one angular degree per frame refresh of the monitor. Participants pressed the “X” key to confirm their estimations. The auditory feedback was then given. There were 40 practice trials where a white feedback line with the actual orientation was displayed after the participant’s response. For the main 400 trials, visual feedback was not given. The task was separated into two blocks, with a break in-between. A post-experiment open-ended questionnaire was given to each participant that probed with increasing specificity whether they had been aware of or could report the probability by location manipulation.

### Post-experiment Questionnaire

After the 400 trials and before debriefing, participants were given a short questionnaire consisting of the following four open-ended questions.

(1) Did anything about the experimental task stand out to you?
(2) Please describe any strategies you may have used.
(3) Did you feel that you perceived some stimuli better or differently than others, or in certain cases? Did you notice any change over time in your experience?
(4) Do you think that some orientations are more likely at certain times? If yes, please elaborate.

### Analysis

Analysis was done using the *R* statistical software package (R Core Team, 2016). Bayesian inference testing was conducted using the BayesFactor R package (Morey et al., 2015). Initiation time (IT) was taken as the time from the appearance of the response line to when the participants’ first adjusted the orientation of response line. This measure was used because it has proven in the past to be robust (Jabar and Anderson, 2015), and since it is uncontaminated by the amount of movement required to report the orientation that is a function of the angle of the stimulus. We also analyzed vacillations, the number of times that participants changed directions when generating their reports. Vacillations might be linked to the confidence of the decision. Confidence is likely post-decisional (e.g., Hilgenstock et al., 2014), and is not tightly coupled to perceptual precision (Jabar and Anderson, 2015; Samaha et al., 2016).

Angular error was measured as the difference (in degrees) between what was presented and what was estimated. The possible range of angular errors occupied an axial space ranging from -90 (anti-clockwise error) to +89° (clockwise error), after which the report wraps back as an error of -90°. We took the median of the absolute magnitude of these errors as our primary dependent variable (see Prinzmetal et al., 1998; Gorard, 2005).

**FIGURE 2:**
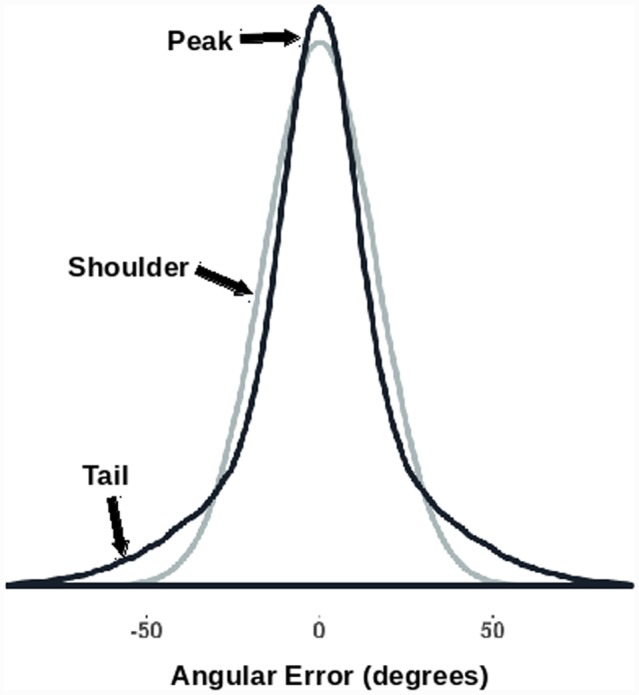
**Kurtosis as a description of the shape of a distribution.** The two generated error distributions have the same median absolute value (10.8°) while having significantly different *excess* kurtoses [darker curve = 1.85; lighter (normal) curve = 0.0]. Note how the distribution with the increased kurtosis has a higher peak, narrower shoulders, and broader tails.

The kurtosis of the (signed) angular error distribution was also looked at. The kurtosis of a distribution is a function of the fourth moment of the data (DeCarlo, 1997). Normally distributed errors would have zero *excess* kurtosis. In previous studies we have found that orientation probability affects kurtosis leading to non-normal error distributions (see Anderson, 2014; Jabar and Anderson, 2015). Higher probability orientations shift the weight from the ‘shoulders’ of the distribution to the ‘tails’ and/or the ‘peak’ of the distribution. This results in a higher computed kurtosis (**Figure 2**). Changes in kurtosis therefore reflect changes in the shape of the error distribution, with different kurtoses suggestive of different types of perceptual errors. For example, if an effect only works to reduce the number of small errors, we should see the shoulders move into the peaks, while the tails (large errors) might stay relatively intact. This should reduce both median absolute error and increase kurtosis. However, if an effect acts uniformly across the whole range of errors, we expect a reduction in the median absolute angular error without a change in the ‘shape’ (kurtosis) of the distribution.

## Results

Data from one participant was removed because the participant failed to respond to most trials. For the remaining nineteen participants, two-way repeated measure ANOVAs were computed for median angular error, angular error kurtosis, mean vacillations, and median ITs. Alpha cut-offs were taken at *p* = 0.05. A summary of the raw results are shown in **Table 1**.

**Table 1:** Summary of Means.

	Spatial Cuing			
	Congruent		Incongruent	
Orientation Probability	High: 80%	Low: 20%	High: 80%	Low: 20%
Vacillations	0.093 (0.071)	0.105 (0.078)	0.088 (0.073)	0.178 (0.091)
Initiation Time (ms)	189 (116)	243 (120)	216 (127)	278 (125)
Median Angular Error (deg)	8.02 (2.49)	8.74 (2.69)	8.63 (2.09)	9.53 (3.00)
Kurtosis	0.84 (1.25)	-0.08 (0.72)	1.61 (1.96)	-0.18 (1.01)

*Values given are means across participants (± 1 SD).*

### Vacillations and Initiation Times

**FIGURE 3:**
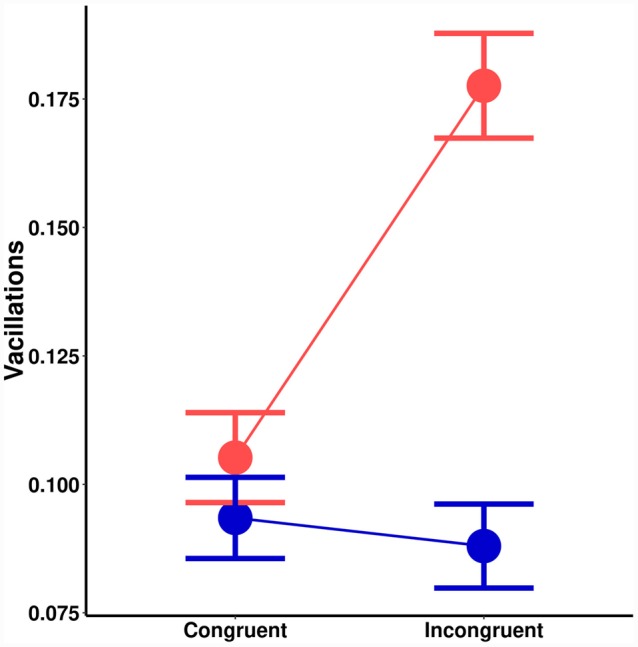
**Effects on vacillations (change in the direction of motion) as a function of spatial cuing (*x*-axis) and orientation probability (blue = high-probability, red = low-probability)**.

There was a significant main effect of orientation probability on vacillations [*F*(1,18) = 9.73, *MSE* < 0.01, *p* = 0.006]. Lower probability led to more vacillations. There was also a significant main effect of spatial cuing [*F*(1,18) = 13.19, *MSE* < 0.01, *p* = 0.002]. Invalid spatial cues led to more vacillations. A significant interaction effect (**Figure 3**) was also noted [*F*(1,18) = 21.48, *MSE* < 0.01, *p* = 0.001]. *Post hoc* tests suggested no effect of spatial cuing on vacillations for high probability orientations [*t*(18) = 0.64, *p* = 0.532], but there was one with low-probability orientations [*t*(18) = 4.70, *p* < 0.001]. Chiefly, congruently cued trials (*M* = 0.11) had less vacillations than incongruent ones (*M* = 0.18).

**FIGURE 4:**
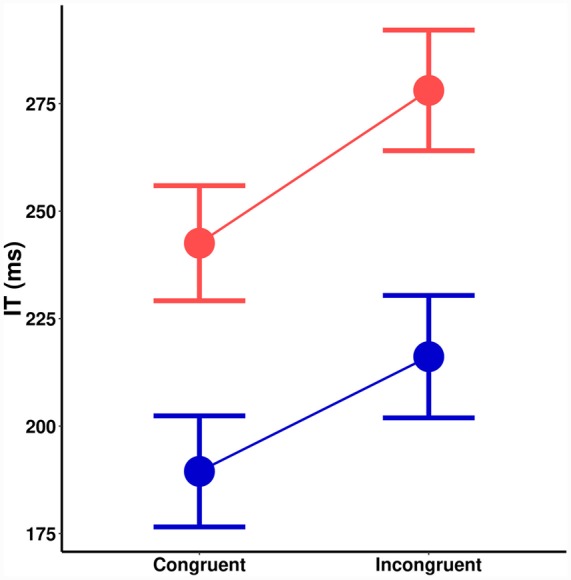
**Initiation time (ms) as a function of spatial cuing (*x*-axis) and orientation probability (blue = high-probability, red = low-probability)**.

A significant main effect of spatial cuing on IT, [*F*(1,18) = 19.62, *MSE* = 938, *p* < 0.001] a significant main effect of orientation probability [*F*(1,18) = 73.48, *MSE* = 855, *p* < 0.001], and no interaction [*F*(1,18) = 0.6, *MSE* = 578, *p* = 0.433] is seen in **Figure 4**. There was no effect of cuing on the size of the orientation probability effect [*t*(18) = 0.80, *p* = 0.433]. Bayesian hypothesis testing returned a Bayes Factor (*BF*) of 0.32 (±0.01%), which is moderate evidence in favor of the *null* hypothesis: That the effects of spatial cuing and stimulus probability are independent. The same trends remained even if trials where participants vacillated in making their responses are removed.

### Median Angular Errors and Kurtosis

**FIGURE 5:**
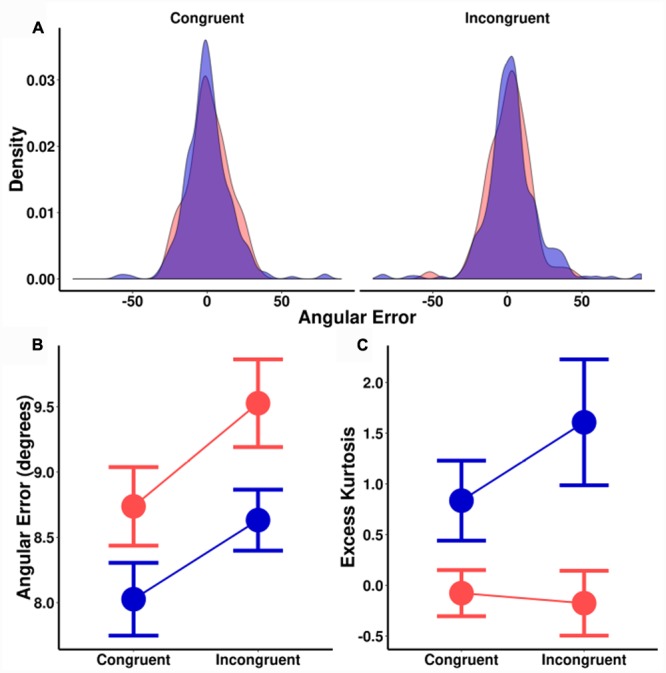
**(A)** Sample distribution of signed angular errors for one participant. Left plot: Congruently cued locations. Right plot: Incongruently cued locations. **(B)** Median angular error (degrees) as a function of spatial cuing (*x*-axis) and orientation probability. **(C)** Kurtosis as a function of spatial cuing (*x*-axis) and orientation probability. (Blue = high-probability, red = low-probability).

The trends are identical for angular errors (**Figure 5B**). There was a significant main effect of spatial cuing on angular precision, [*F*(1,18) = 5.2, *MSE* = 1.8, *p* = 0.035], a significant main effect of orientation probability [*F*(1,18) = 4.9, *MSE* = 2.5, *p* = 0.039], and no significant interaction [*F*(1,18) = 0.1, *MSE* = 1.5, *p* = 0.744]. As before, there was no effect of cuing on the size of the orientation probability effect [*t*(18) = 0.33, *p* = 0.744], with a corresponding *BF* of 0.25 (±0.02%). This is moderate evidence in favor of the null hypothesis. In short, while both spatial cuing *and* orientation probability significantly impacted ITs *and* perceptual precision, these effects were additive.

For kurtosis (**Figures 5A,C**), there was a significant main effect of orientation probability [*F*(1,18) = 19.9, *MSE* = 1.7, *p* < 0.001] without an effect for spatial cuing [*F*(1,18) = 0.3, *MSE* = 1.6, *p* = 0.260], nor a significant interaction effect [*F*(1,18) = 2.2, *MSE* = 1.6, *p* = 0.155]. Trials that were spatially cued congruent and incongruent to the Gabor location were compared separately for each probability condition, with Bayesian testing again revealing evidence for the null hypothesis [high-probability *BF* = 0.60 (±1%); low-probability *BF* = 0.25 (±2%)]. In contrast, high and low-probability trials were compared separately for both congruent and incongruently cued trials, [congruent *BF* = 7.0 (±1%); incongruent *BF* = 15.4 (±1%)]: Strong evidence in favor of the alternative hypothesis. These results suggest that while orientation probability causes a change in the shape (kurtosis) of the error distributions, spatial cueing does not. As with previous studies, this kurtosis effect by orientation probability was not an artifact of an uneven number of trials. Sub-sampling and bootstrapping with matched numbers of trials found similar effects on kurtosis.

### Post-experiment Questionnaire

For the post-experiment questionnaire, no participant reported that probability was being manipulated, even when directly asked “Do you think that some orientations are more likely at certain times? If yes, please elaborate.” This was the same as our previous experiments using these probabilities for orientation estimation tasks. Some participants volunteered that they found the task more difficult when ‘incorrectly’ cued.

## Discussion

Probability and spatial cuing manipulations generally result in similar behavioral effects: Speed and accuracy are facilitated (Anderson and Druker, 2013; Anderson, 2014; Jabar and Anderson, 2015, in press). As both these effects resemble what might traditionally be labeled as ‘attentional,’ one could surmise a common mechanism.

We manipulated both types of cues conjointly within a single perceptual estimation task. Effects from previous reports where either one or the other manipulation was done were replicated. Both manipulations improved ITs *and* perceptual precision. However, the manipulations act independently: There were *additive* effects in *both* ITs and perceptual precision. If these two manipulations share the same mechanism, one would expect only high-probability orientations *in the cued space* to benefit. Instead, the size of the orientation probability effect was unaffected by the spatial cue. Further, spatial cuing did not affect the shape (kurtosis) of the angular error distribution while orientation probability did (and as has been shown previously Anderson, 2014; Jabar and Anderson, 2015). In addition, a report that predated our discovery of kurtosis effects (Anderson and Druker, 2013) had used spatial cues. We reanalyzed those data here and found, consistent with this report, that spatial cuing alone does not affect kurtosis. Had the same perceptual mechanisms been invoked by both spatial cuing and orientation probability, we would have expected common effects on error distribution shape. Instead our results suggest that spatial cuing and orientation probability affect perceptual precision and speed of response via different mechanisms.

What might these mechanisms be? We have previously suggested (Jabar and Anderson, 2015, in press) that exposure to orientation probability results in neural tuning, i.e., changes in the range of orientations in which neurons respond to. Tuning changes occur when *features* are cued (David et al., 2008; Paltoglou and Neri, 2012; Çukur et al., 2013; Ling et al., 2015). Tuning changes also occur when there is no explicit cue. Ringach et al. (1997) and Schoups et al. (2001) showed that when monkeys receive orientation training, the tuning width of V1 neurons selective for the trained orientations are ‘sharpened.’ This is consistent with the suggestion that learnt likelihoods are reflected in the early phase of sensory processing (Summerfield and Egner, 2009). We argue that V1 neurons selective for probable orientations are ‘sharpened’ when exposed to orientation probability, which modulates feedforward perceptual processing (Jabar et al., submitted).

Although V1 neural responses to the subsequent target are also modulated by exogenous spatial cues (Wang et al., 2015), spatial manipulations are thought to recruit a gain rather than a tuning mechanism (e.g., Ling et al., 2009; Carrasco, 2011). Spatial manipulations are also necessarily non-selective for features. Unlike with orientation probability (Ringach et al., 1997) or feature-based cues (Martinez-Trujillo and Treue, 2004) where only the feature-relevant neurons are facilitated, spatial cues provide no information about the *features* of the upcoming target stimuli. These differences in feature-based and space-based mechanisms could account for the independent effects of orientation probability and spatial exogenous cuing, respectively. Whereas orientation probability might affect precision through selectivity tuning neurons preferring the probable orientations, exogenous spatial cuing could be recruiting a non-selective gain process that increases apparent contrast either by increasing the input baseline of neural responses (Cutrone et al., 2014) or by speeding up information accrual (Carrasco and McElree, 2001; Carrasco et al., 2004).

If the two cue-types modulate perceptual precision through separate perceptual mechanisms, how can we understand the interaction on vacillations? It might be important to note that there could be multiple processing stages (Sternberg, 1969) involved in perceptual discrimination (e.g., Sun and Landy, 2016), and what we might be observing are additive effects of exogenous spatial cuing and orientation probability in the *stimulus processing stage*, but interactions in the downstream response or *decision-making stage.* If vacillations are indicative of the confidence associated with the decision-making process (e.g., Petrusic and Baranski, 2009), then they are not necessarily tied to the quality of perceptual processing. Precision-based probability effects were seen even across trials equated for self-reported confidence (Jabar and Anderson, 2015). Perhaps in the congruently cued case, participants were explicitly expecting targets in the ‘cued’ location, which reduced the impact that probability had on their confidence? It could be that *repeats* in orientation are driving confidence more than probability *per se*. It is important that future studies seeking to understand the differences between the two mechanisms not simply equate perception with detection, since the act of decision-making involves additional factors.

An outstanding issue is why spatial exogenous cuing affects orientation precision while spatial probability does not (Jabar and Anderson, in press). While they are both space-based, one critical difference is that the presence of an explicit visual cue activates V1 neurons *prior* to the target occurrence (e.g., Wang et al., 2015). Speculatively, this might cause V1 neurons to be more excitable in the target phase. The lack of an explicit cue also places orientation probability more closely to orientation training (e.g., Ringach et al., 1997) than to trial-by-trial feature-based cuing, which has been argued to recruit both gain *and* tuning mechanisms (Ling et al., 2009). What the overlap is between orientation probability and orientation cuing is an open question that perhaps could be addressed with a similar study to the current one.

If orientation probability effects are distinct from other attentional manipulations, should they still be called ‘attentional’? If attention is understood as a class of *effects* (Anderson, 2011), then that label still applies. Where confusion arises is when attention is understood as a general *causal* mechanism. Orientation probability mechanisms are distinct from spatial exogenous cuing mechanisms. They lead to independent effects, and we suggest that this is due to orientation probability causing neural tuning differences. Whether that also applies for other attentional manipulations is an open question. It is worth keeping in mind that effects that look behaviorally similar are not necessarily indicative of common neural and psychological mechanisms.

## Ethics Statement

This study was carried out in accordance with the recommendations of the Declaration of Helsinki with written informed consent from all subjects. The protocol was approved by the University of Waterloo’s Office of Research Ethics.

## Author Contributions

SJ did the experiment design, data collection, data analysis, and the writing of this report. BA supervised the research and contributed to the experiment design and writing of the report.

## Conflict of Interest Statement

The authors declare that the research was conducted in the absence of any commercial or financial relationships that could be construed as a potential conflict of interest.

## Footnotes

1 http://www.freesound.org/people/tombola/sounds/49219/
